# Description of Lifestyle, Including Social Life, Diet and Physical Activity, of People ≥90 years Living in Ikaria, a Longevity Blue Zone

**DOI:** 10.3390/ijerph18126602

**Published:** 2021-06-19

**Authors:** Romain Legrand, Gilles Nuemi, Michel Poulain, Patrick Manckoundia

**Affiliations:** 1 “Pôle Personnes Âgées”, Hospital of Champmaillot, University Hospital, 21079 Dijon, France; legrand.rom1@gmail.com; 2 Department of Biostatistics and Bioinformatics, François Mitterrand Hospital, University Hospital, 21079 Dijon, France; gilles.nuemi@chu-dijon.fr; 3 Institute for the Analysis of Change in Historical and Contemporary Societies (IACCHOS), Université Catholique de Louvain, 1348 Ottignies-Louvain-la-Neuve, Belgium; michel.poulain@uclouvain.be; 4 Estonian Institute for Population Studies, Tallinn University, 10120 Tallinn, Estonia; 5 INSERM U-1093, Cognition, Action and Sensorimotor Plasticity, University of Burgundy Franche-Comté, 21000 Dijon, France

**Keywords:** healthy ageing, Ikaria, longevity, oldest old aged people

## Abstract

A cross-sectional observational study was conducted to describe the lifestyle of people ≥90 years, living in Evdilos or Raches, two municipalities of the Greek island of Ikaria, classified a longevity blue zone. The 71 participants were interviewed and underwent the Mediterranean Islands study food frequency questionnaire (MEDIS-FFQ) and the international physical activity questionnaire (IPAQ). The frequency of social contacts was daily for 77.9% of participants, weekly for 16.1%, and monthly for 5.9%. Most participants (90.0%) believed in God, and 81.4% took part in religious events. A total of 62.0% attended Panigiria festivals. Access to primary health care was considered difficult in the past for 66.2% of participants, while 22.1% felt that it remained difficult at the time of the survey. The level of adherence to the Mediterranean diet was 62.7% (61.6% in women and 64.0% in men). Physical activity levels were moderate or high for 71.8% of participants (59.5% of women and 85.3% of men). In conclusion, the participants had a very high level of family solidarity, social interaction and physical activity. The results concerning the Mediterranean diet are less convincing. It would be interesting to study the impact of these factors on the longevity of the oldest old aged people living in Ikaria.

## 1. Introduction

Populations around the world are rapidly ageing. The number of people aged ≥60 will rise from 12% to 22% of the total global population between 2015 and 2050 [1]. Whether this demographic aging represents an opportunity depends heavily on health status in old age, and this is why the World Health Organization (WHO) has made “healthy ageing” one of its priorities for 2020–2030 [1].

To better understand the meaning of healthy ageing, it may be interesting to study populations whose longevity is particularly high. In 2000, Poulain and Pes identified a location in Sardinia with exceptional longevity. This region has been designated as a longevity blue zone (LBZ) [2], a concept that has been defined in a previous study [3].

Ikaria is a mountainous island located in the Eastern Aegean Sea between Mykonos and Samos. Despite a low socioeconomic status and the presence of cardiovascular risk factors (hypertension, diabetes, and tobacco smoking), the elderly inhabitants of this tiny island have maintained a relatively good health status, in particular a low rate of depression, high muscle strength, and good functional abilities [3,4].

A long and healthy lifespan can be explained by both modifiable and non-modifiable factors. Lifestyle is one important modifiable factor that can impact life expectancy. We thus performed this study in order to describe the lifestyle, including social life, diet, and physical activity, of the inhabitants of Ikaria aged 90 and over. Some previous work has focused on the Ikaria LBZ [2,3,4], but they studied a much younger population and did not investigate the same aspects, in a particular social lifestyle.

## 2. Methods

### 2.1. Design

From 21 October 2012 to 21 October 2013, we conducted a cross-sectional observational study on potential factors influencing healthy ageing in people aged ≥90 living in Evdilos and Raches, the two municipalities located in the northwestern part of the island, where the longevity has been found to be the highest [3,5].

### 2.2. Population

Potential participants aged ≥90 were identified due to the lists established by Poulain and Pes in 2009 [5]. We updated these lists by consulting the local population registers that were available in the municipal offices of Evdilos and Raches and by visiting each village in these municipal units. The list was completed by involving participants from the survey of Panagiotakos and colleagues [4]. This study was conducted in accordance with the Declaration of Helsinki and National standards. An Ethics Committee was consulted. It was not opposed to this study, which was a simple observational survey among volunteers who did not have patient status and with no modification in their usual management.

### 2.3. Questionnaire

In addition to the data analyzed in our previous publication [3], the questionnaire included the following items:

- Social data: (1) Frequency of contact with other people: never, monthly, weekly, and daily; (2) Religion: religious belief, participation in religious ceremonies, and religious fasting; (3) Panigiria: participation and opinion; (4) Politics: participation in political events and opinion on the communist party; (5) Primary health care access during childhood and currently; (6) Radioactive hot springs: access in the past and effect on health if applicable; (7) Swimming in the Icarian Pelagos, the sea around the island, and swimming cessation age if applicable; (8) Daytime naps: did the participant take daytime naps and how long if applicable.
- The level of adherence to the Mediterranean diet was assessed using the Mediterranean Islands study (MEDIS)-food frequency questionnaire (MEDIS-FFQ). This questionnaire was evaluated and validated for older adults living in Mediterranean areas [6]. It measures the frequency of consumption of 15 categories of various food groups and beverages that are usually consumed in Mediterranean countries during a regular week: meat, fish, milk, yogurt and other dairy, fruits, vegetables, greens, salads, legumes, cereals, sweets, and olive oil. It evaluates the size of the portion usually consumed (small, regular, or large) and includes some specific information about the type of bread, type of spirits, use of other added fats (margarine, sterols, stanols, and butter), type of cheese, and coffee and tea consumption (type and quantity of cups per day). Food frequency is expressed as follows: daily, weekly (1–2 and 3–5 times/week), monthly (2–3 times/month), rarely, or never. Level of adherence to the Mediterranean diet was evaluated by calculating the Mediterranean Diet Score (MedDietScore) [7]. The score varies from 0 to 55, the highest values indicating a better adhesion. Five classes of adhesion have been defined, each corresponding to a different risk of having a coronary heart disease [8]. We added a question about the origin of the food and the wine consumed by the oldest old aged people (OO) in Ikaria (local or supermarket).
- The level of physical activity was assessed using the international physical activity questionnaire (IPAQ) in its short 7-item form validated in Greek [9]. The questionnaire consisting of six questions in order to record the number of days (frequency) and the number of minutes (min) per day (min/day) (duration) of participation on all kinds of vigorous, moderate, and walking physical activities during the last seven days. In addition, a seventh question records the time spent sitting during an average weekday [10]. According to the recommendations of the IPAQ committee, a physical activity score was calculated expressed in MET-min per week (min/week), and the respondents were classified into three physical activity categories: low, moderate and high [11].

### 2.4. Statistical Analysis

Categorical variables (social data, except age of cessation of Icarian Pelagos and midday nap duration, diet data, except MedDietScore, and categorical score of the IPAQ) were described as numbers and percentages, while quantitative variables (age of cessation of Icarian Pelagos, midday nap duration, MedDietScore, and continuous score and time spent sitting of the IPAQ) were expressed as means or medians with standard deviation or interquartile ranges.

Women and men were compared according to the frequency of social contacts, religious belief and participation, fasting, positive opinion and participation in Panigiria, positive opinion on the communist party and participation in political events, difficulty accessing primary health care, hot springs access in the past, swimming in the Icarian Pelagos and age of cessation, midday napping, origin of food and wine consumed, MedDietScore, categorial and continuous scores of the IPAQ, and time spent sitting. 

In this study, which aimed to describe the lifestyle of this particular population, categorical variables were compared using the chi-squared test or the Fisher’s test, when appropriate. According to the result of the normality test, the Student’s unpaired *t*-test or the Wilcoxon rank sum test was used for quantitative variables. Statistical significance was accepted for *p* < 0.05.

Normality was assessed using the Shapiro–Wilk test.

R statistical tools embedded in RStudio software, version 1.3.1093, was used to conduct all statistical analyses. All statistical tests were two sided [12].

## 3. Results

Of the 98 eligible subjects, we included 71. Among the non-participants, 21 were not seen mostly because they were temporarily overseas, one refused to take part and five died during the survey. There was not a significant difference between the 71 participants and the 27 non-participants in terms of age (*p* = 0.380) or sex (*p* = 0.285).

### 3.1. Social Characteristics of Participants

The 71 included participants were interviewed and examined at their place of residence. Results on help proxy, number of years spent on the island, residence, occupation, family status, and family environment are detailed in our previous article. 

Regarding social contact, 77.9% of participants reported daily frequency. Ninety percent of participants believed in God, 34.3% of them participated in monthly religious events, and 32.9% participated yearly. A total of 54.9% did not practice Greek Orthodox fasting. A total of 62.0% participated in Panigiria and 92.1% had a positive opinion of this event. A total of 39.4% of participants took part in political events and 37.9% had a positive opinion of the communist party. The access to primary health care was considered difficult in the past for 66.2% of participants, while 22.1% felt that it remained difficult at the time of the survey. A total of 29.6% of participants went to the radioactive hot springs in the south of the island, and, among them, 83.3% thought it had a positive effect on their health. A total of 79.7% went to swim in the Icarian Pelagos and the average age at which they stopped swimming was 75.5 ± 19.0 years old. Overall, 69.6% reported taking a daytime nap, more men (79.4%) than women (60.0%), and the average was of 80.6 ± 43.7 min each day. These results are detailed in Table 1.

### 3.2. Adherence to the Mediterranean Diet

The MedDietScore was 34.5 ± 3.7 for the whole population, 33.9 ± 4.0 in women, and 35.2 ± 3.4 in men. The relative odds of having coronary heart disease were 1.42 in women and 1 in men (Table 2).

As concerns the origin of food, 32.9% of participants consumed locally, 31.4% bought their food from the supermarket, and 35.7% did both. Regarding wine, it was 96.4% locally produced when consumed (Table 1).

### 3.3. Physical Activity 

The overall total physical activity score median was 1022 ± 3066 MET-min/week in women and 3066 ± 4768.5 MET-min/week in men. The levels of physical activity were moderate or high for 71.8% of participants (59.5% of women and 85.3% of men). The median value of time spent sitting was 60 min/day in women and 90 min/day in men (Table 3).

## 4. Discussion

This study is very interesting because it analyzed the lifestyle of very old adults living in the Ikaria LBZ. 

Among the previous articles that focused on the Ikaria LBZ, that of Poulain et al. was more devoted to the description of the geography of Ikaria [2]. Panagiotakos et al. were interested in the lifestyle of the population of Ikaria, with a focus on older adults [4]. While their work was similar to our study, differences include the target population, which was significantly older in our study (10 years older) and the social parameters collected, which were more numerous in our study. In addition to the social parameters common to the two studies, we added: religion practice, politics participation or opinion, radioactive hot springs, swimming in the Icarian Pelagos, and origin of food consumed. Finally, our present study focused on the lifestyle (social life, diet, and physical activity) of OOs, while our previous study focused on sociodemographic data, medical profile, households, and working career [3].

The main strength of this study is the fact that every participant was visited and the survey was performed personally by the main investigator. Accordingly, the precision of the answers was enhanced, and we were able to get a closer look at each participant’s way of life. Although eligible for this study, 27 of the OOs did not participate, mostly because they were temporarily overseas. Since no statistical difference was found between participants and individuals who were not included, the study population was representative of the officially recognized population of OOs in the municipal units of Raches and Evdilos.

Most of the participants had spent their whole life in Ikaria. This is interesting because it suggests that they have been exposed to the same environmental factors, such as diet for practically their whole lives.

The fact that many OOs lived in an isolated house is due to the particular organization of the dwellings on the island, which are scattered. This model, which is extremely rare in the rest of Greece, makes it possible for each house to have a garden or land to cultivate. It also explains why the shops are closed during the day in Ikaria and only open in the evening, after the agricultural work is done [13]. The most common occupation on Ikaria was farming, not fishing as might be expected, and the median retirement age indicated that a significant proportion of OOs continued to work. In our study, we found that a higher proportion of women were affected by widowhood compared to men, who were mostly married. This is consistent with other data on partnership status in the OOs [14]. Studies have shown the influence of marital status on health. Indeed, mortality is higher among widowers, whatever their sex, but the association varies by sex, and widowhood is more detrimental in men than women [15,16]. Among men, marriage could protect from depressive symptoms and partly explain the low rates of depression assessed in Ikaria [17]. Despite the high level of widowhood among women, only 27% lived alone compared with 55% in the MEDIS sample [18]. Household size for women and men was almost the same (i.e., 2.3 ± 1.5), mainly due to the presence of a family member, most often a child, living in the same place or nearby. Family solidarity may contribute to increased feelings of well-being, and reduced distress and cognitive impairments among the OOs in Ikaria without a spouse [19]. This could also partially explain why, in our survey, very few OOs were in a nursing home. Whatever their sex, participants had a high level of social interaction. Most of them (i.e., 78%) saw their neighbors, family, or friends every day. Social interaction has beneficial effects on health, improving the prognosis of cardiovascular and neurovascular diseases, such as myocardial infarction and stroke, reducing mortality risk, and improving mental health [20]. One of the original aspects of our study is the analysis of the participants’ involvement in religion. We found that a significant proportion of OOs reported believing in God and attending religious celebrations. Most often, religious participation involved important religious events such as Easter, Christmas, weddings, or funerals. Several studies have found a positive correlation between religious belief and practice, and mental and physical health and longevity [21,22]. This is explained more by behavioral than psychological factors [23]. Religious participation contributes to a reduction in unhealthy behaviors (alcohol abuse, smoking, unhealthy diet, etc.) and increases social support [23]. In Greece, Orthodox Christianity recommends three main periods of fasting per year, which can be characterized as a vegetarian diet including fish and seafood [24]. However, in our study, few OOs followed these fasting periods. Originally, the Panigiria were gatherings related to religious events in Greek Orthodox Christianity. Currently, the Panigiria remain major events in Ikaria, especially their festive aspect. From May to September, Panigiria are organized by volunteers almost every week in the different villages of the island. The meat consumed is often wild goat native to Ikaria, and the wine is local. Participants are invited to dance together on the “Kariotiko” [25]. The money collected is used to finance improvements in the villages such as building roads and/or schools, supplying water to villages [25]. The Panigiria provide the inhabitants of Ikaria with opportunities to come together and celebrate, and they also serve the general interest [25].

We found that the vast majority of OOs had a positive opinion on Panigiria and some continued to take part, often in the one organized in their village. Participation in Panigiria could have a positive influence on the health of the OOs by increasing their social integration and by encouraging physical activity linked to dance [26]. 

One of the new aspects of our study is the fact that we were also interested in the politics participation or opinion of OOs living in the Ikaria LBZ. Another particularity of Ikaria is its relationship with the communist party, which earned it the nickname of “Red Island”. This is in part due to the fact that the island served as a land of exile for many communist political prisoners during the Greek civil war [27]. However, less than half of the OOs expressed an interest in the communist party (Kommounistiko Komma Elladas (KKE)) and most never attended political events. 

In our study, the majority of participants felt that they had no difficulty accessing primary health care (i.e., 78%). Primary medical follow-up is an important means of detecting illness and ensuring better control of chronic diseases. It may also lead to lower chronic disease mortality [28].

Ikaria is characterized by the presence of radioactive hot springs that flow into the sea to the southeast of the island. These springs expose visitors and swimmers to particularly high levels of radioactivity without reaching the thresholds known to have carcinogenic effects [29]. In our study, few OOs went to radioactive hot springs but many of them went to swim in the Icarian Pelagos, the sea around the island, until old age. Moreover, they spent a large part of their life exposed to radiation of terrestrial origin, which is higher in the western and northwestern parts of the island than in the eastern part [30]. Thus, one of the new aspects of this study is the fact that we assessed whether the OOs living in the Ikaria LBZ visited the radioactive hot springs. Indeed, some studies suggest the existence of a beneficial effect of ionizing radiation according to a hormesis effect [31]. However, this has never been proven; the health effect of low doses of ionizing radiation therefore remains controversial.

The majority (70%) of OOs in Ikaria took a daytime nap, but it is difficult to draw a conclusion from this result. Indeed, some studies associate daytime napping with an increase in mortality from myocardial infarction, while others report a decrease [32,33]. It seems that nighttime sleep duration the most important factor, with a U-shaped association between sleep duration and mortality [34]. Results regarding sleep duration in our previous work suggest that there is a lower risk of all-cause mortality, especially cardiovascular-related mortality [3].

Level of adherence to the Mediterranean diet was good, slightly higher than that found in the MEDIS sample of OOs [18]. In our study, the level of adherence to the Mediterranean diet was associated with protection from coronary heart disease, regardless of the common cardiovascular risk factors. That protection was better and more complete in men. Furthermore, as found in our previous work, the population studied had very few cardiovascular risk factors. Indeed, the rates of diabetes and dyslipidemia were low (19.7% and 12.7% respectively), as was alcohol consumption (27% did not consume it and 63% consumed 1–2 glasses/day) and the smoking (56%, over 94% of women had never smoked, and only 7% of participants smoked at the time of this survey) [3]. In addition, in the present study, 72% of the population had a moderate or high level of physical activity. However, 70% of the population had a history of hypertension, which was probably controlled by diet or antihypertensive treatment seeing as blood pressure was normal at the time of the study [3]. In addition, more than half of the population was overweight or obese (body mass index ≥ 25 kg/m^2^) [3]. The idea that Mediterranean populations are protected from cardiovascular disease through their eating habits comes from the work led by Keys in the 1950s–1980s. In Keys’ work, the seven country study, the Greek and Italian cohorts presented far fewer deaths from cardiovascular disease than other populations living outside the Mediterranean area [35]. Later, in the 1990s, the Lyon diet heart study showed a protective role of the Mediterranean diet in the secondary prevention of myocardial infarction [36]. Subsequently, the protective effects of the Mediterranean diet have been demonstrated in overall mortality, cardiovascular disease mortality, cancer incidence or mortality, and incidence of Parkinson disease and Alzheimer disease [37]. The Mediterranean diet is characterized by a high intake of vegetables (legumes, fruits, nuts, and cereals) and olive oil. Fish intake, dairy products (cheese or yogurt), and wine consumption are moderate. Red meat consumption, pastries, and saturated lipids are low [38]. Keys also emphasized the importance of its social aspect by associating the concepts of celebration and sharing [39]. Unfortunately, today, given the change in the food production system and globalization, the healthy traditional eating habits found in the Mediterranean diet are threatened [40]. In Ikaria, a significant proportion of OOs consumed locally produced food, often their own production. It is likely that their level of adherence to the Mediterranean diet remained good because the island has retained some of its traditions and people have land around their homes on which they can grow their own produce.

In 2007, public health recommendations were drawn up on the physical activity of older adults. They recommend performing moderate-intensity aerobic (endurance) physical activity such as a 30-min walk five days/week or vigorous-intensity aerobic activity for a minimum of 20 min three days/week [41]. These recommendations, corresponding to the second category (moderate) of the IPAQ, were therefore followed by 72% of the OOs included in our study. Few studies have focused specifically on physical activity in the elderly. Data indicate that approximately 65% of those aged 75 years and older do not meet recommended levels of regular physical activity [42]. The benefits of regular physical activity in older adults are extensive. Indeed, physical activity reduces the risk of cardiovascular diseases, hypertension, type 2 diabetes, osteoporosis, obesity, colon cancer, breast cancer, chronic obstructive pulmonary disease, anxiety, and depression and/or has a substantial therapeutic role on these diseases [43,44,45]. Of particular importance to older adults, physical activity reduces risk and injury from falls, and prevents or delays cognitive impairment and disability [46,47,48]. Finally, physical activity improves sleep quality [49]. In Ikaria, the recommendations concerning physical activity were more followed by men (85.3%) than women (59.4%). This is consistent with previous studies on physical activity in the OOs and could explain why we found in a previous study a higher prevalence of cardiovascular risk factors (hypertension, diabetes, and dyslipidemia) and cardiovascular diseases in women [3,42]. Physical activity increases longevity by reducing all-cause mortality, mainly cardiovascular and respiratory [50]. It is a more powerful predictor of mortality among men than other established risk factors for cardiovascular diseases [51]. The good results concerning the physical activity of OO men in Ikaria could therefore explain their good health status despite the relatively high prevalence of risk factors such as hypertension, diabetes, and tobacco [3]. In Ikaria, the physical activity of the OOs consisted mainly in farming activities and walking (to go to work or the villages). In the villages, the participants would often meet with friends at the “kafenio” or continue an activity in one of the village stores. The walk was often on uneven ground because of the mountainous nature of the island.

Our study has some limitations such as the number of participants included, which was relatively small but exhaustive. Moreover, though our study was population-based, some OOs in the target area could not be surveyed because they were temporally absent, creating a potential selection bias. In our study, some data were self-reported, which may create an information bias. Another limitation is the physical activity assessment with the IPAQ, which was validated in subjects aged 18–65 years. However, it is the only validated physical activity assessment questionnaire that we found in Greek.

## 5. Conclusions

Living longer makes sense if we are in good health. This is what is called “healthy ageing” by WHO, who has made it a priority for the decade to come. In this context, after reporting on the medical profile, cardiovascular risk factors, household, and working career of OOs living in the Ikaria LBZ in our previous article, here we described the lifestyle of this same population. We found social factors to be very important, with a high level of family solidarity and very little institutionalization. The level of social interaction was also very high since the majority of OOs maintained daily social contacts. Many still participated in social events (religious, Panigiria) and some continued to work, helping to maintain good social integration. The level of adherence to the Mediterranean diet was good, but not exceptional, enabled in part by local food production. Finally, the level of physical activity was very good, especially among men, mainly because they continue to do agricultural work. We believe it would be worthwhile to conduct a study in order to assess the impact of these three main factors on the longevity of OOs living in Ikaria.

## Figures and Tables

**Table 1:** Social data of participants.

Parameter		Women	Men	Total
		*n/N* (%) or Mean ± SD	*n/N* (%) or Mean ± SD	*n/N* (%) or Mean ± SD
Frequency of social contacts	Never	0	0	0
	Monthly	3/34 (8.8)	1/34 (2.9)	4/68 (5.9)
	Weekly	4/34 (11.8)	7/34 (20.6)	11/68 (16.1)
	Daily	27/34 (79.4)	26/34 (76.5)	53/68 (77.9)
Religious belief		33/36 (91.7)	30/34 (88.2)	63/70 (90.0)
Religious participation	Never	6/37 (16.2)	7/33 (21.2)	13/70 (18.6)
	<1/Month	13/37 (35.1)	10/33 (30.3)	23/70 (32.9)
	>1/Month	11/37 (29.7)	13/33 (39.4)	24/70 (34.3)
	>1/Week	7/37 (18.9)	3/33 (9.1)	10/70 (14.3)
Religious fasting	Never	16/37 (43.2)	23/34 (67.6)	39/71 (54.9)
	<5 days/year	4/37 (10.8)	2/34 (5.9)	6/71 (8.5)
	5–15 days/year	8/37 (21.6)	5/34 (14.7)	13/71 (18.3)
	>15 days/year	9/37 (24.3)	4/34 (11.8)	13/71 (18.3)
Positive opinion on Panigiria		28/32 (87.5)	30/31 (96.8)	58/63 (92.1)
Participation in Panigiria	Never	16/37 (43.2)	11/34 (32.4)	27/71 (38.0)
	<1/month	17/37 (45.9)	17/34 (50.0)	34/71 (47.9)
	>1/month	3/37 (8.1)	6/34 (17.6)	9/71 (12.7)
	>1/week	1/37 (2.7)	0	1/71 (1.4)
Positive opinion on the Communist Party		8/28 (28.6)	14/30 (46.7)	22/58 (37.9)
Participation in political events	Never	23/37 (62.2)	20/34 (58.8)	43/71 (60.6)
	<1/month	11/37 (29.7)	11/34 (32.4)	22/71 (31.0)
	>1/month	3/37 (8.1)	3/34 (8.8)	6/71 (8.5)
	>1/week	0	0	0
Primary health care access difficulty	Childhood	23/35 (65.7)	22/33 (66.7)	45/68 (66.2)
	Nowadays	6/35 (17.1)	9/33 (27.3)	15/68 (22.1)
Hot spring access in the past		11/37 (29.7)	10/34 (29.4)	21/71 (29.6)
Icarian Pelagos	Swimming	27/36 (75.0)	28/33 (84.8)	55/69 (79.7)
	Age Of Cessation	74.0 ± 18.6	76.8 ± 19.7	75.5 ± 19.0
Midday nap	Presence	21/35 (60.0)	27/34 (79.4)	48/69 (69.6)
	Duration (Minutes)	78.9 ± 26.9	81.9 ± 54.0	80.6 ± 43.7
Origin of food consumed	Local	13/36 (36.1)	10/34 (29.4)	23/70 (32.9)
	Supermarket	9/36 (25.0)	13/34 (38.2)	22/70 (31.4)
	Mixed	14/36 (38.9)	11/34 (32.4)	25/70 (35.7)
Origin of wine consumed	Local	23/24 (95.8)	30/31 (96.8)	53/55 (96.4)
	Supermarket	1/24 (4.2)	1/31 (3.2)	2/55 (3.6)
	Mixed	0	0	0

*n*: number of subjects for each category parameter; *N*: number of subjects of group population; SD: standard deviation.

**Table 2:** Results of the Mediterranean Islands study food frequency questionnaire (MEDIS-FFQ).

Parameter	Women *N* = 36	Men *N* = 32	Total *N* = 68	p *
	Mean ± SD or OR	Mean ± SD or OR	Mean ± SD or OR	
MedDietScore (range 0–55)	33.9 ± 4.0	35.2 ± 3.4	34.5 ± 3.7	0.146
Coronary heart disease	1.42	1	1.42	-

*N*: number of subjects of group population; MedDietScore: Mediterranean diet score; SD: standard deviation; OR: odds ratio. * *p* value for comparison between women and men.

**Table 3:** Results of the physical activity assessment using the international physical activity questionnaire (IPAQ).

Parameter		Women *N* = 37	Men *N* = 34	Total *N* = 71
		*n* (%) or Median ± IQR	*n* (%) or Median ± IQR	*n* (%) or Median ± IQR
Categorical Score	Low	15 (40.5)	5 (14.7)	20 (28.2)
	Moderate	10 (27.0)	9 (26.5)	19 (26.8)
	High	12 (32.4)	20 (58.8)	32 (45.1)
Continuous Score (MET-min/week)		1022 ± 3066	3066 ± 4768.5	1533 ± 3924
Time spent sitting (min/day)		60 ± 240	90 ± 120	90 ± 150

*N*: number of subjects of group population; *n*: number of subjects for each category parameter; IQR: interquartile ranges; MET: metabolic equivalent of task; min: minute(s).

## Data Availability

The data presented in this study are available on request from the corresponding author. The data are not publicly available.

## References

[1] World Health Organization Ageing and Life Course. http://www.who.int/ageing/en/.

[2] Poulain M., Pes G.M., Grasland C., Carru C., Ferrucci L., Baggio G., Franceschi C., Deiana L. (2004). Identification of a geographic area characterized by extreme longevity in the Sardinia island: The AKEA study. Exp. Gerontol..

[3] Legrand R., Manckoundia P., Nuemi G., Poulain M. (2019). Assessment of the Health Status of the Oldest Olds Living on the Greek Island of Ikaria: A Population Based-Study in a Blue Zone. Curr. Gerontol. Geriatr. Res..

[4] Panagiotakos D.B., Chrysohoou C., Siasos G., Zisimos K., Skoumas J., Pitsavos C., Stefanadis C. (2011). Sociodemographic and Lifestyle Statistics of Oldest Old People (>80 Years) Living in Ikaria Island: The Ikaria Study. Cardiol. Res. Pract..

[5] Poulain M., Pes G. (2009). Report of the Validation of the Exceptional Longevity in Ikaria-Unpublished Internal Report.

[6] Tyrovolas S., Pounis G., Bountziouka V., Polychronopoulos E., Panagiotakos D.B. (2010). Repeatability and Validation of a Short, Semi-Quantitative Food Frequency Questionnaire Designed for Older Adults Living in Mediterranean Areas: The MEDIS-FFQ. J. Nutr. Elder..

[7] Panagiotakos D.B., Pitsavos C., Stefanadis C. (2006). Dietary patterns: A Mediterranean diet score and its relation to clinical and biological markers of cardiovascular disease risk. Nutr. Metab. Cardiovasc. Dis..

[8] Panagiotakos D.B., Milias G.A., Pitsavos C., Stefanadis C. (2006). MedDietScore: A computer program that evaluates the adherence to the Mediterranean dietary pattern and its relation to cardiovascular disease risk. Comput. Methods Prog. Biomed..

[9] Papathanasiou G., Georgoudis G., Papandreou P., Spyropoulos P., Georgakopoulos D., Kalfakakou V., Evangelou A. (2009). Reliability measures of the short International Physical Activity Questionnaire (IPAQ) in Greek young adults. Hellenic. J. Cardiol..

[10] Craig C.L., Marshall A.L., Sjöström M., Bauman A.E., Booth M.L., Ainsworth B.E., Pratt M., Ekelund U., Yngve A., Sallis J.F. (2003). International physical activity questionnaire: 12-country reliability and validity. Med. Sci. Sports Exerc..

[11] (2005). Guidelines for Data Processing and Analysis of the International Physical Activity Questionnaire (IPAQ)-Short and Long Forms. http://www.IPAQ.ki.se.

[12] R Core Team (2019). R: A Language and Environment for Statistical Computing.

[13] Γιαννίρη H. (1998). Παρόν και μέλλον. Τα προβλήματα και οι δυνατότητες οικονομικής ανάπτυξης της Ικαρίας. H Καθημερινή..

[14] Aging Stats-Older Americans 2016. https://agingstats.gov/.

[15] Moon J.R., Kondo N., Glymour M.M., Subramanian S.V. (2011). Widowhood and Mortality: A Meta-Analysis. PLoS ONE.

[16] Shor E., Roelfs D.J., Curreli M., Clemow L., Burg M.M., Schwartz J.E. (2012). Widowhood and mortality: A meta-analysis and me-ta-regression. Demography.

[17] Lee G.R., DeMaris A., Bavin S., Sullivan R. (2001). Gender Differences in the Depressive Effect of Widowhood in Later Life. J. Gerontol. Ser. B.

[18] Tourlouki E., Polychronopoulos E., Zeimbekis A., Tsakountakis N., Bountziouka V., Lioliou E., Papavenetiou E., Polystipioti A., Metallinos G., Tyrovolas S. (2009). The ’secrets’ of the long livers in Mediterranean islands: The MEDIS study. Eur. J. Public Health.

[19] Okabayashi H., Liang J., Krause N., Akiyama H., Sugisawa H. (2004). Mental health among older adults in Japan: Do sources of social support and negative interaction make a difference?. Soc. Sci. Med..

[20] Seeman T.E. (1996). Social ties and health: The benefits of social integration. Ann. Epidemiol..

[21] Alves R.R.D.N., Alves H.D.N., Barboza R.R.D., Souto W.D.M.S. (2010). The influence of religiosity on health. Ciênc. Saúde Coletiva.

[22] Powell L.H., Shahabi L., Thoresen C.E. (2003). Religion and spirituality. Linkages to physical health. Am. Psychol..

[23] Zhang W. (2008). Religious participation and mortality risk among the oldest old in China. J. Gerontol. Ser. B.

[24] Sarri K.O., Linardakis M.K., Bervanaki F.N., Tzanakis N.E., Kafatos A.G. (2004). Greek Orthodox fasting rituals: A hidden characteristic of the Mediterranean diet of Crete. Br. J. Nutr..

[25] Πάσχαρη-Κουλουλία A. (1998). Τα καριώτικα πανηγύρια. Εχουν μακρά παράδοση, λατρευτικό και ψυχαγωγικό χαρακτήρα και κοινωφελή σκοπό. H Καθημερινή..

[26] Mavrovouniotis F.H., Argiriadou E.A., Papaioannou C.S. (2010). Greek traditional dances and quality of old people’s life. J. Bodyw. Mov. Ther..

[27] Papalas A.J. (2005). Rebels and Radicals. Icaria 1600–2000.

[28] Graves B.A. (2008). Integrative Literature Review: A Review of Literature Related to Geographical Information Systems, Healthcare Access, and Health Outcomes. Perspect. Health Inf. Manag..

[29] Florou H., Trabidou G., Nicolaou G. (2007). An assessment of the external radiological impact in areas of Greece with elevated natural radioactivity. J. Environ. Radioact..

[30] Trabidou G., Florou H. (2010). Estimation of dose rates to humans exposed to elevated natural radioactivity through different pathways in the island of Ikaria, Greece. Radiat. Prot. Dosim..

[31] Tubiana M. (2009). Prevention of cancer and the dose-effect relationship: The carcinogenic effects of ionizing radiations. J. Soc. Fr. Radiother. Oncol..

[32] Bursztyn M., Stessman J. (2005). The Siesta and Mortality: Twelve Years of Prospective Observations in 70-Year-Olds. Sleep.

[33] Naska A., Oikonomou E., Trichopoulou A., Psaltopoulou T., Trichopoulos D. (2007). Siesta in healthy adults and coronary mortality in the general population. Arch. Intern. Med..

[34] Gallicchio L., Kalesan B. (2009). Sleep duration and mortality: A systematic review and meta-analysis. J. Sleep Res..

[35] Keys A., Ancel K. (1980). Seven Countries: A Multivariate Analysis of Death and Coronary Heart Disease.

[36] de Lorgeril M., Salen P., Martin J.L., Monjaud I., Delaye J., Mamelle N. (1999). Mediterranean diet, traditional risk factors, and the rate of cardiovascular complications after myocardial infarction: Final report of the Lyon Diet Heart Study. Circulation.

[37] Sofi F., Cesari F., Abbate R., Gensini G.F., Casini A. (2008). Adherence to Mediterranean diet and health status: Meta-analysis. BMJ.

[38] Trichopoulou A., Costacou T., Bamia C., Trichopoulos D. (2003). Adherence to a Mediterranean diet and survival in a Greek population. N. Engl. J. Med..

[39] Keys A., Keys M. (1975). How to Eat Well and Stay Well. The Mediterranean Way.

[40] Da Silva R., Bach-Faig A., Quintana B.R., Buckland G., De Almeida M.D.V., Serra-Majem L. (2009). Worldwide variation of adherence to the Mediterranean diet, in 1961–1965 and 2000–2003. Public Health Nutr..

[41] Nelson M.E., Rejeski W.J., Blair S.N., Duncan P.W., Judge J.O., King A.C., Macera C.A., Castaneda-Sceppa C., American College of Sports Medicine, American Heart Association (2007). Physical activity and public health in older adults: Recommendation from the American College of Sports Medicine and the American Heart Association. Circulation.

[42] Paganini-Hill A., Kawas C.H., Corrada M.M. (2011). Activities and mortality in the elderly: The Leisure World cohort study. J. Gerontol. A Biol. Sci. Med. Sci..

[43] Kokkinos P., Sheriff H., Kheirbek R. (2011). Physical inactivity and mortality risk. Cardiol. Res. Pract..

[44] Kruk J., Czerniak U. (2013). Physical Activity and its Relation to Cancer Risk: Updating the Evidence. Asian Pac. J. Cancer Prev..

[45] Sjösten N., Kivelä S.-L. (2006). The effects of physical exercise on depressive symptoms among the aged: A systematic review. Int. J. Geriatr. Psychiatry.

[46] Tinetti M.E., Baker D.I., McAvay G., Claus E.B., Garrett P., Gottschalk M., Koch M.L., Trainor K., Horwitz R.I. (1994). A multifac-torial intervention to reduce the risk of falling among the elderly people living in the community. N. Engl. J. Med..

[47] Laurin D., Verreault R., Lindsay J., MacPherson K., Rockwood K. (2001). Physical Activity and Risk of Cognitive Impairment and Dementia in Elderly Persons. Arch. Neurol..

[48] Landi F., Abbatecola A.M., Provinciali M., Corsonello A., Bustacchini S., Manigrasso L., Cherubini A., Bernabei R., Lattanzio F. (2010). Moving against frailty: Does physical activity matter?. Biogerontology.

[49] Hood B., Bruck D., Kennedy G. (2004). Determinants of sleep quality in the healthy aged: The role of physical, psychological, circadian and naturalistic light variables. Age Ageing.

[50] Paffenbarger R.S., Hyde R.T., Wing A.L., Hsieh C.C. (1986). Physical activity, all-cause mortality, and longevity of college alumni. N. Engl. J. Med..

[51] Myers J., Prakash M., Froelicher V., Do D., Partington S., Atwood J.E. (2002). Exercise Capacity and Mortality among Men Referred for Exercise Testing. N. Engl. J. Med..

